# Engineered bacterial hydrophobic oligopeptide repeats in a synthetic yeast prion, [*REP-PSI*^+^]

**DOI:** 10.3389/fmicb.2015.00311

**Published:** 2015-04-21

**Authors:** Fátima Gasset-Rosa, Rafael Giraldo

**Affiliations:** Department of Cellular and Molecular Biology, Centro de Investigaciones Biológicas – Consejo Superior de Investigaciones CientíficasMadrid, Spain

**Keywords:** amyloid cross-seeding, prion variants/strains, RepA-WH1 prionoid, [*REP-PSI*^+^] prion, *Saccharomyces cerevisiae*, synthetic biology

## Abstract

The yeast translation termination factor Sup35p, by aggregating as the [*PSI*^+^] prion, enables ribosomes to read-through stop codons, thus expanding the diversity of the *Saccharomyces cerevisiae* proteome. Yeast prions are functional amyloids that replicate by templating their conformation on native protein molecules, then assembling as large aggregates and fibers. Prions propagate epigenetically from mother to daughter cells by fragmentation of such assemblies. In the N-terminal prion-forming domain, Sup35p has glutamine/asparagine-rich oligopeptide repeats (OPRs), which enable propagation through chaperone-elicited shearing. We have engineered chimeras by replacing the polar OPRs in Sup35p by up to five repeats of a hydrophobic amyloidogenic sequence from the synthetic bacterial prionoid RepA-WH1. The resulting hybrid, [*REP-PSI*^+^], (i) was functional in a stop codon read-through assay in *S. cerevisiae*; (ii) generates weak phenotypic variants upon both its expression or transformation into [*psi*^-^] cells; (iii) these variants correlated with high molecular weight aggregates resistant to SDS during electrophoresis; and (iv) according to fluorescence microscopy, the fusion of the prion domains from the engineered chimeras to the reporter protein mCherry generated perivacuolar aggregate foci in yeast cells. All these are signatures of *bona fide* yeast prions. As assessed through biophysical approaches, the chimeras assembled as oligomers rather than as the fibers characteristic of [*PSI*^+^]. These results suggest that it is the balance between polar and hydrophobic residues in OPRs what determines prion conformational dynamics. In addition, our findings illustrate the feasibility of enabling new propagation traits in yeast prions by engineering OPRs with heterologous amyloidogenic sequence repeats.

## Introduction

**FIGURE 1:**
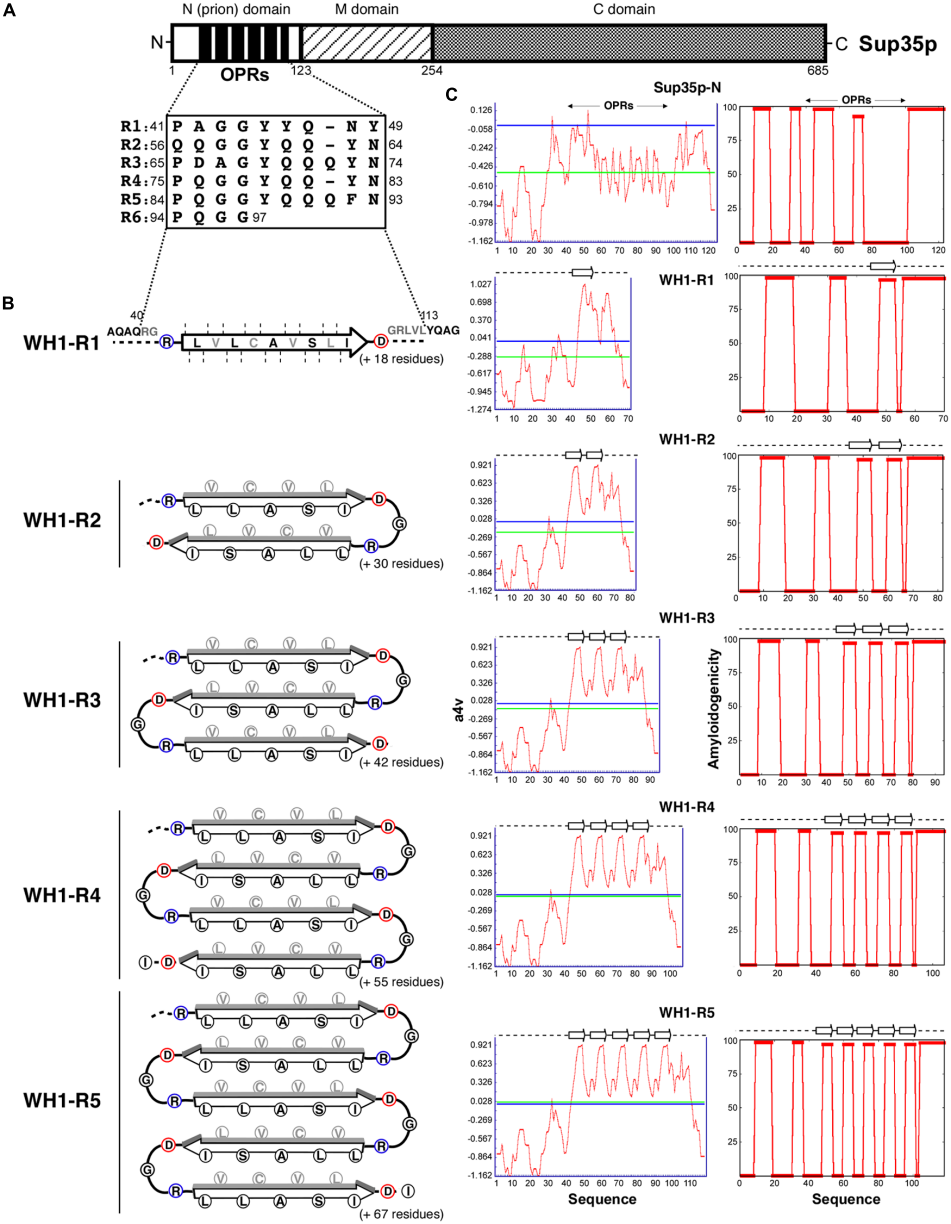
**Chimeras between the yeast prion protein Sup35p and the amyloidogenic stretch in the bacterial prionoid RepA-WH1. (A)** Schematic representation of *Saccharomyces cerevisiae* eRF3 (Sup35p), with its domain composition and the N-terminal 5½ OPRs highlighted. **(B)** The R0 + WH1-R1-5 chimeras. Sup35p OPRs were totally replaced by 1–5 tandem repeats of the hydrophobic amyloidogenic stretch in the RepA-WH1 variant A31V (arrow; Giraldo, 2007). The heterologous sequences were engineered to fold as zigzag β-arcades (reviewed in Kajava et al., 2010), in which turns were made by inserting a Gly residue between the natural Asp (blue) C- and Arg (red) ‘gatekeeper’ N-ends from contiguous WH1 repeats. β-strand packing (faces of strands in black, backs in gray) would be guided by interdigitation of chemically compatible side chains. β-arcades from distinct protein monomers would stack through main-chain hydrogen bonds into a parallel superpleated β-structure, as proposed for Sup35p/[*PSI*^+^] (reviewed in Kajava et al., 2010). In the resulting fiber, the engineered repeats should have to accommodate as a hydrophobic spine within an otherwise polar Q/N-rich axis. **(C)** The amyloidogenicity profiles (red plots) for the distinct chimeric N-domains, as estimated by the AGGRESCAN (left column; Conchillo-Solé et al., 2007) and WALTZ (right; Maurer-Stroh et al., 2010) algorithms, suggest that the synthetic peptide arrays assemble as amyloids. Horizontal lines: blue, aggregation hot spot threshold; green, sequence average.

Modularity is a basic principle of organization in proteins and their assemblies. In the case of the aggregation-prone amyloidogenic proteins, modularity comes from the existence of sequence stretches that become the β-strand building blocks in cross-β sheets (reviewed in Eisenberg and Jucker, 2012). Such stretches can either have hydrophobic or polar average residue compositions, but usually not both at the same time, thus posing constrains to homotypic interactions, which require chemically compatible side chains to cross-aggregate. Polar glutamine/asparagine (Q/N)-rich amyloidogenic sequences are a hallmark of prion domains in yeast (Alberti et al., 2009; reviewed in Liebman and Chernoff, 2012), in which cross-seeding has been well characterized, e.g., for the Rnq1p/[*PIN*^+^] prion nucleating the aggregation of Sup35p/[*PSI*^+^] (Vitrenko et al., 2007; Sharma and Liebman, 2013). In the translation releasing factor Sup35p, its prion-forming domain (N) includes up to five and a half oligopeptide repeats (OPRs; **Figure 1A**; reviewed in Tessier and Lindquist, 2009). OPRs, while still assembled in the amyloid fibers, have been proposed to be the targets for the disaggregase chaperone Hsp104p (Chernoff et al., 1995) that, with the aid of Hsp70 chaperones Ssa1-4p (Shorter and Lindquist, 2008; Winkler et al., 2012), generate [*PSI*^+^] propagons, i.e., oligomeric primordia that are readily diffusible to the progeny (Cox et al., 2003; Derdowski et al., 2010). The modularity of Sup35p OPRs has allowed their partial or total replacement by heterologous sequences, such as the octapeptide repeats in the mammalian prion protein PrP (Parham et al., 2001; Dong et al., 2007; Tank et al., 2007), while keeping the resulting chimeras their original function as epigenetic determinants of reading-through stop codons in yeast. With the accumulated knowledge on Sup35p/[*PSI*^+^], this is probably the most suitable model system to address the molecular determinants of the prion condition in proteins (Sabate et al., 2015).

RepA, the DNA replication protein of the *Pseudomonas* plasmid pPS10, is one of the members best characterized of a protein family spread across a large group of plasmids from Gram-negative bacteria (reviewed in Giraldo and Fernández-Tresguerres, 2004). Soluble RepA dimers, which function as transcriptional self-repressors of the *repA* gene, undergo a large conformational change upon binding to specific DNA sequences from the plasmid replication origin, resulting in their dissociation into replication-competent RepA monomers (Díaz-López et al., 2003, 2006). Such structural change consists in an increase in β-sheet at the expense of the α-helical secondary structure component and affects WH1, the most N-terminal of the two ‘winged-helix’ domains in RepA, which is transformed from a dimerization domain into a secondary DNA binding module, ancillary to the main DNA recognition determinant, C-terminal WH2 (Giraldo et al., 1998, 2003). RepA monomers are aggregation-prone and, once a replication round is completed, the resulting plasmid copies are held together through interactions between RepA monomers bound to the replication origins of the two plasmid molecules, thus inhibiting further replication rounds (Gasset-Rosa et al., 2008a). Such complex interplay between transcriptional repression and DNA replication initiation/inhibition, focused further research on the molecular basis for the ligand (DNA)-modulated balance between RepA solubility and functional aggregation. In RepA-WH1, a single hydrophobic sequence stretch partially folded as a α-helix changes its conformation, upon transient binding to specific DNA sequences, to assemble as a β-strand in amyloid fibers (Giraldo, 2007; Gasset-Rosa et al., 2008b). The RepA-WH1 domain, when expressed in *Escherichia coli* as a metastable fusion to the fluorescent protein mCherry, behaves as a synthetic prionoid, i.e., an amyloidogenic protein lacking infectivity (Aguzzi, 2009). Unlike prions in yeast, RepA-WH1 triggers a sort of intracellular amyloid ‘proteinopathy’ in its host (Fernández-Tresguerres et al., 2010), where the ‘vertical’ propagation (i.e., from mother cell to daughter cells) and the conformational selection of ‘strains’ of the prionoid are dependent on the Hsp70 chaperone DnaK, rather than on the Hsp104 ortholog ClpB (Gasset-Rosa et al., 2014). A recent electron microscopy reconstruction of the RepA-WH1 fibers, as templated *in vitro* on soluble molecules of the protein by amyloid seeds preformed *in vivo*, showed that the fibers are composed of intertwined amyloid tubules built by distorted protein monomers (Torreira et al., 2015).

Since the amyloidogenic sequence stretch in RepA-WH1 has clearly distinct features to those naturally found in yeast prions, – i.e., a single hydrophobic amyloidogenic stretch in a properly folded domain, rather than multiple repeats with polar residue composition in the frame of an intrinsically disordered tail – the bacterial prionoid is a suitable source of radically heterologous sequences to explore their ability to supplant the OPRs in Sup35p/[*PSI*^+^]. We report here that chimeras between Sup35p and at least three repeats of the hydrophobic amyloidogenic stretch in RepA-WH1 constitute novel functional prions in yeast that we have named [*REP-PSI*^+^]. Furthermore, [*REP-PSI*^+^] can cross-seed the propagation of [*PSI*^+^], but generating [*PSI*^+^]*^WH1^*, a new strain with very low mitotic stability (i.e., epigenetically unstable), as expected for a weak prion with severe limitations on the generation of propagons. These findings validate the possibility of tailoring yeast epigenetics by engineering aggregation-prone hydrophobic amyloidogenic repeats within prion-forming domains.

## Materials and Methods

### Plasmid Constructs

#### Substitution of Sup35p-OPRs by Tandem Repeats of the Amyloidogenic Stretch in RepA-WH1 (WH1-R1-5)

The reporter plasmid pUKC1620 (Parham et al., 2001; Von der Haar et al., 2007), harboring a WT copy of the *SUP35* gene with its natural promoter, was used as template for PCR-amplification (*Pfu* DNApol) of NΔ*R6*-*sup35* using primers with BamHI (3′ to the promoter) and EcoRV (annealing after the 5th repeat) ends. After digestion with those enzymes, the product replaced the BamHI-EcoRV *N-SUP35* fragment in the original plasmid, leading to pUKC_*R5*. On this vector, *WH1-R1* was inserted through PCR, including the reverse primer (5′-CGAGgatatcC**GATCAAAGACACAGCGCATAGCACTAG**ATTGAATTGCTGCTGAT), after the EcoRV site (lowercase), the complement of the sequence coding for the nine core amino-acid residues (LVLCAVSLI) in WH1(A31V) (bold; Giraldo, 2007). *WH1-R2* was built in a similar way, but the reverse primer had that sequence repeated twice with a TCC/Gly spacer. For pUKC_*R0*, a construction lacking the five natural OPRs in Sup35p, the PstI site previous to the first OPR in the parental plasmid was mutated to EcoRV (QuikChange kit, Stratagene), followed by digestion with this enzyme and in-frame religation of the vector. *WH1-R1-3* were built by annealing the pre-phosphorylated (T4-PNK) oligos 5′-GGCCGCCTAGTGCTATGC**GCTGTGTCTTTGATCGAT**/5′-GCATAGCACTAGGCGGCC**ATCGATCAAAGACACAGC** (complementary sequences in bold) followed by filling of the ends (Klenow DNApol) and self-ligation, a step that generated an extra terminal half repeat. Each repeat in the concatemers includes the natural C-terminal Asp and N-terminal Arg found in the RepA-WH1 amyloidogenic stretch (Giraldo, 2007) plus a linker Gly. Upon ligation of the concatemers mix at the EcoRV site in pUKC_*R0* and transformation into *E. coli*, DNA sequencing identified clones with up to three RepA-WH1 repeats. Since *WH1-R4-5* were recalcitrant to be obtained through such approach, a fragment including three RepA-WH1 repeats was made through chemical synthesis (ATG:biosynthetics, Germany) and inserted into pUKC_*R1-2*, in which EcoRV had been regenerated by site directed mutagenesis, to give pUKC_*R4-5*.

#### Plasmids for Protein Expression in Yeast under the pGAL1 Promoter

The full-length *WH1*(*Rn*)*-SUP35* chimeras were inserted into the pYeF2 vector (Cullin and Minvielle-Sebastia, 1994) after amplification by PCR, using as templates the pUKC series (see above) and primers having BamHI and NotI ends. These chimeras carried a C-terminal hemagglutinin (HA)-tag from the vector. For fluorescence microscopy observation, fusions were made between the NM-domains from the chimeras and the monomeric red fluorescent protein mCherry at their C-termini. With this purpose, each NM-domain was amplified by PCR on the pUKC plasmids, with oligonucleotides carrying BamHI and BspEI ends, and then ligated with a BspEI (5′)-NotI (3′) mCherry fragment, amplified using as template pWH1(A31V)-mRFP (Fernández-Tresguerres et al., 2010). The BamHI-NotI fragments were then cloned into pYeF2 as above.

#### Plasmids for Protein Expression in *E. coli*

The fusions of *NM-WT*, *NM-R0*, *NM-R0* + *WH1-R2-4* to mCherry were cloned into the pRG vectors (*Ptac*, His_10_ N-tag; Fernández-Tresguerres et al., 2010) by PCR amplification, performed with primers with SacII (5′-NM) and XbaI (3′-mCherry) ends. All constructs were verified by DNA sequencing.

### *Saccharomyces cerevisiae* Strains

For screening prion-dependent translation through stop codons, it was used a derivative of the strain 74D-694: *MAT****a*** *ade1-14*^UGA^ *trp1-289 his3-Δ200 ura3-52 leu2-3,112 sup35::loxP* [pYK810] [*PSI*^+^] [*PIN*^+^]. For fluorescence microscopy, the YJT28 strain (*ΔARS305::kanMX*, *ade2-1::ADE2*, W303-1a) was selected to suppress autogenous red fluorescence.

### Epigenetic Assay for Yeast Colony Color

The pUKCs derivatives (encoding the different chimeric alleles) were electroporated in a [*PSI*^+^] [*PIN*^+^] strain (see above) that initially carried pYK810, a plasmid bearing a copy of *SUP35* (Von der Haar et al., 2007). For displacing the resident pYK810, thus assuring that the incoming pUCKs were the only source of Sup35p (or its chimeras with the WH1 repeats), colonies growing in SD-His were replicated on the same medium containing 0.1% 5-FOA, thus counter-selecting for cells that carried pYK810 (*URA3*). Colonies were then plated on ¼YPD and SD-adenine. For full development of color in colonies, agar plates were incubated for ≥72 h at 30∘C and then transferred to 4∘C for 24 h before photographic documentation.

### Protein Aggregate Transformation into [*PSI*^-^] Cells

Overnight cultures inoculated from pUKCs-carrying red [*psi*^-^] colonies, that have spontaneously lost the [*PSI*^+^] phenotype, were diluted ⅛ into 60 ml of YPD and grown to OD_600_ = 0.5. Cells were washed with water and 1 M sorbitol, resuspended in SCE buffer (1 M sorbitol, 100 mM Na-citrate pH 5.8, 10 mM EDTA, 10 mM DTT, 2 mg/ml lyticase), and then incubated at 30∘C for 1 h. The resulting spheroplasts were centrifuged and resuspended in SCT buffer (1 M sorbitol, 10 mM Tris^.^HCl pH 7.5, 10 mM CaCl_2_) and 100 μl were co-transformed with 2 μM of pYeF2 (Tanaka, 2010) as a marker (*URA3*), 10 μg of carrier ssDNA and 5 μl of whole cell extracts (i.e., the low-speed supernatants after cells lysis, see below) from pUKCs/[*REP-PSI*^+^] cells. Suspensions were incubated at room temperature (RT) with rotation mixing for 30 min. Then 44% PEG4000, 10 mM Tris^.^HCl pH 7.5, 10 mM CaCl_2_ buffer was added and further incubated for 45 min. Spheroplasts were then sedimented, resuspended in SOS medium (1 M sorbitol, 25% YPD, 7 mM CaCl_2_), added to 10 ml of top agar (SD-URA, 2% dextrose, 0.8% agar, 1 M sorbitol, 2% YPD), and platted on SD-URA agar. Incubation proceeded for ≥72 h at 30∘C. Large size colonies were selected and spotted on ¼YPD agar. To address the stability of [*PSI*^+^], white colonies obtained after transformation of Sup35p-WT [*psi*^-^] cells with R0 + WH1-R3-5 protein extracts were grown in YPD at 30∘C overnight. They were then diluted to OD_600_ = 0.001, grown for 24 h, and 30 μl plated on ¼YPD agar and incubated as above. Transformation and stability assays were performed independently twice.

### Aggregate Extraction and Sedimentation Assay

Two hundred ml cultures of yeast carrying the full-length protein chimeras cloned into pYeF2s (see above) were grown overnight in selective medium (SD-Ura) with glucose. Then cultures were diluted to OD_600_ = 0.07 in SD-Ura, but with 2% raffinose and 0.1% glucose, and grown to OD_600_ = 0.2, when protein expression was induced by adding 2% galactose and further incubated until OD_600_ = 2. Cells were then harvested and resuspended in 500 μl of 25 mM Tris^.^HCl pH 6.8, 250 mM NaCl, 5 mM EDTA, 10% glycerol (plus protease inhibitors; Roche). Lysis was then carried-out with glass beads (Lysing matrix C) in a MP FastPrep-24 homogenizer (five cycles, level 5, for 30 s at 4∘C). Cell debris was removed by a low-speed sedimentation step (600 × *g*, 3 min). Two hundred microliter of the resulting whole cell extracts were ultracentrifuged at 50,000 rpm (100,000 × *g*), for 15 min at 4∘C (Beckman Optima Max-XP, TLA100 rotor). Supernatants were collected and pellets were resuspended in 200 μl of the lysis solution. Proteins in equivalent volumes of supernatant and pellet fractions were analyzed by SDS-PAGE (10% polyacrylamide; 30 μg/lane) plus Western-blotting, using an anti-HA antibody (Roche, 1:1,000) and chemiluminescence detection (ECL2; Pierce).

### Semi-Denaturing Detergent Agarose Gel Electrophoresis (SDD-AGE)

Total cell lysates (45 μl, at 30 mg/ml) from yeast having the chimeras expressed from pYeF2 (see above) were mixed with 15 μl of loading buffer (TAE 2X, 20% glycerol, 8% sarkosyl, 0.5 g/l bromophenol blue, plus protease inhibitors). Samples were incubated at RT for 10 min, and electrophoresis performed in 1.5% agarose gels (TAE 1X, 0.1% SDS) at 100 V for 7.3 h, 10∘C (Molina-García and Gasset-Rosa, 2014). Proteins were then transferred to a PVDF membrane in a Trans-Blot device (Bio-Rad) in TAE 1X, 0.1% SDS, at 16 V for 15 h, 10∘C. Detection was performed with anti-HA (1:1,000).

### Visualization of Aggregates by Fluorescence Microscopy

#### Overexpression of the NM-mCherry Chimeras

pYeF2s encoding the chimeras were transformed into the YJT28 strain and protein expression was carried out as described above. Culture aliquots were taken along 22 h for live cell observation.

#### Fluorescence Microscopy

It was performed with a Nikon Eclipse 90i microscope, equipped with CFI PLAN APO VC (NA 1.40) oil immersion objective and a Hamamatsu ORCA-R^2^ CCD camera. A red filter with excitation 543/22 and emission 593/40 was used. Differential interference contrast (DIC) images were also captured.

### Purification of His_10_-NM-mCherry Chimeras

Protein expression and purification were performed as described for His_6_-RepA-WH1 (Giraldo, 2007), but extending the Ni^2+^-IMAC gradient to 0.5 M imidazole. Protein stocks (30 μM) were kept at -70∘C in 0.1 M Na_2_SO_4_, 20 mM Na_2_HPO_4_ pH 6, 5 mM 2-mercaptoethanol, 10% glycerol.

### Amyloid Assembly of NM-mCherry Chimeras *In Vitro*

Protein chimeras (15 μM) were assembled *in vitro* by still incubation at 5∘C for a month, as described for RepA-WH1 (Giraldo, 2007), in 0.1 M Na_2_SO_4_, 60 mM Hepes pH 8, 8 mM MgSO_4_, 14% PEG4000, 6% MPD. Samples were examined in a JEOL JEM-1230 electron microscope.

### Circular Dicroism (CD) Spectroscopy

Spectra of the purified NM-mCherry chimeras (15 μM) were acquired in 0.1 M Na_2_SO_4_, 15 mM Na_2_HPO_4_ pH 6, at 5∘C, as described (Giraldo, 2007).

### Analytical Ultracentrifugation

NM-mCherry chimeras were dialyzed in 0.1 M Na_2_SO_4_, 20 mM Na_2_HPO_4_ pH 6, 5 mM 2-mercaptoethanol. Four hundred microliter of each sample were diluted to 0.8, 0.2, and 0.08 mg/ml and then centrifuged for 5 min at 13,000 rpm, 4∘C. The clarified supernatants were studied by sedimentation velocity. Centrifugation was carried out in a Beckman–Coulter XLI analytical ultracentrifuge, at 48,000 rpm and 20∘C, measuring absorbance at 280 nm. Sedimentation coefficient distributions, c(s), were determined, with a confidence level of 0.68, using the SEDFIT 14.1 software (Schuck, 2000).

## Results

### Engineering Chimeras between the Amyloidogenic Stretch in RepA-WH1 and [*PSI*^+^]

Structural modeling of the amyloid fibers assembled by the N-domains of yeast prion proteins suggests that their basic building block might be a β-arch (reviewed in Kajava et al., 2010), in which adjacent Q/N-rich stretches would assemble as β-strands interdigitated through compatible side chains, while the intervening sequence would form a turn. If multiple stretches were present, as in Sup35p OPRs, β-arches would be further folded as β-arcades. The stacking of β-arcades from distinct protein monomers, stabilized through parallel main-chain hydrogen bonding, would result in a parallel superpleated β-structure (reviewed in Kajava et al., 2010). With a β-arcade based model in mind, we used a plasmid reporter system (Parham et al., 2001) in which all the OPRs in Sup35p were replaced by up to five tandem repeats of the hydrophobic amyloidogenic stretch found in the bacterial prionoid RepA-WH1 (Giraldo, 2007; WH1-R1-5; **Figure 1**).

### RepA-WH1 + Sup35p Chimeras are Functional in Yeast

**FIGURE 2:**
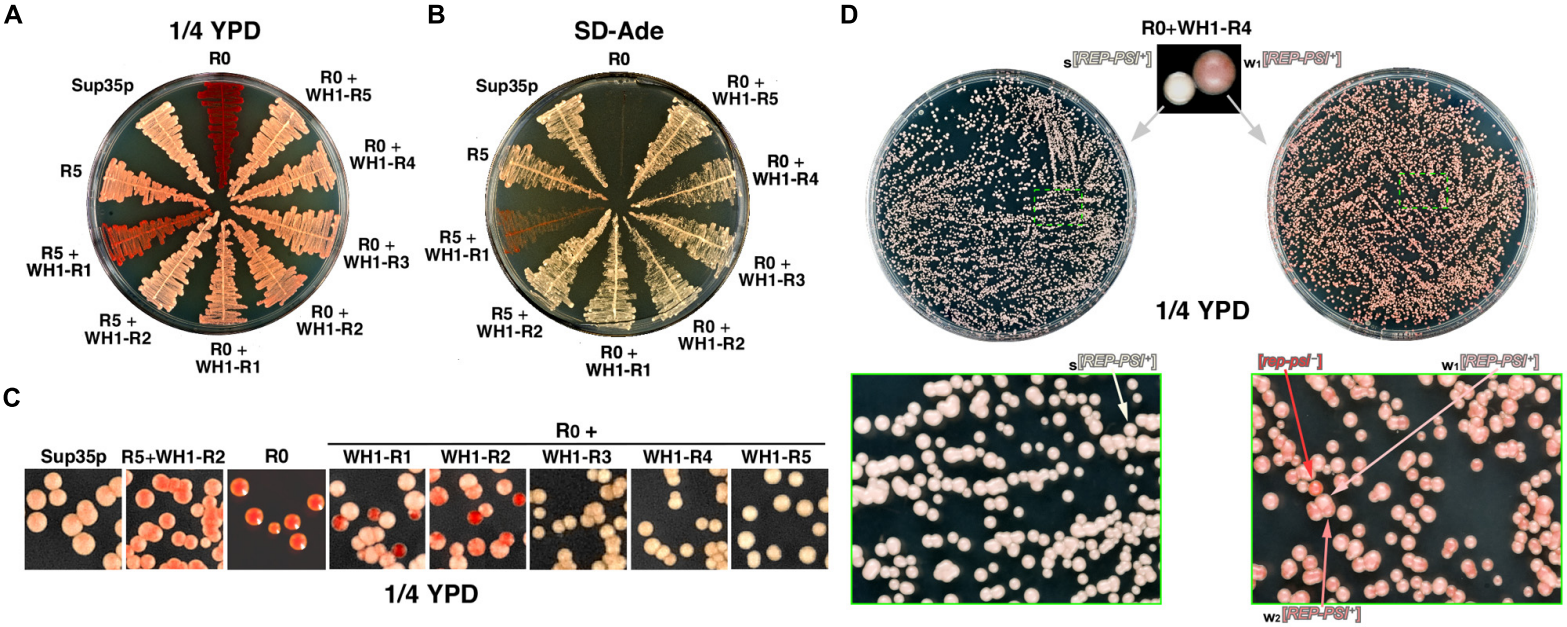
**WH1-Rn/Sup35p chimeras constitute a novel functional prion, [*REP-PSI*^+^]. (A)** A yeast strain carrying the *ade1-14* reporter was transformed with the chimeras (**Figure 1B**). Transformants were streaked on ¼YPD and grown at 30∘C for ≥72 h. Except for R5 + WH1-R1, all the other chimeras restore a [*PSI*^+^]-like color phenotype. **(B)** Same clones as in **(A)** were plated on selective dropout medium lacking adenine (SD-Ade). A [*PSI*^+^]-like stop codon read-through phenotype was confirmed for the chimeras, although very weak for R5 + WH1-R1. **(C)** After several passages in YPD, clones in **(A)** were plated on ¼YPD (top) unveiling the instability (red sectored colonies) of constructs carrying ≤2 WH1 repeats. **(D)** Phenotype variability and stability of the [*REP-PSI*^+^] prion exemplified by the R0 + WH1-R4 chimera. Starting from a single white colony, the prion stably propagates as the strong variant s[*REP-PSI*^+^] (left). However, if the inoculum was seeded from a light pink clone (right), assigned to a weak variant w1[*REP-PSI*^+^], the prion generates with high frequency colonies of a second weaker variant, w2[*REP-PSI*^+^], with a darker tone of pink, besides exhibiting a sensible frequency of prion loss (red colonies, [*rep-psi^-^*]). Green boxes show magnifications of plate sectors in which the relevant colony phenotypes can be appreciated.

The engineered chimeras were expressed in yeast from the *SUP35* promoter in a centromeric plasmid, and tested in an epigenetic red–white colony color assay (**Figure 2**): upon Sup35p aggregation as [*PSI*^+^], the reporter *ade1-14* allele, including a premature amber stop codon, is read-through by the ribosomes allowing for the synthesis of adenine, thus giving white color colonies on rich, unselective medium. Otherwise, soluble Sup35p efficiently terminates translation resulting in red [*psi^-^*] colonies by accumulation of the adenine precursor metabolite 5′-P-ribosyl-5-aminoimidazole (reviewed in Tessier and Lindquist, 2009). In the absence of endogenous Sup35p-WT, achieved in [*PSI*^+^] cells upon displacement of a resident *SUP35* plasmid by vectors encoding the chimeras, five of the native OPRs fused to two chimeric RepA-WH1 repeats (R5 + WH1-R2) were sufficient to yield a [*PSI*^+^]-like prion phenotype. However, with just one bacterial repeat (R5 + WH1-R1) the phenotype of this chimera was even weaker (more intense red color) than the R5 parental, suggesting that the insertion of a single hydrophobic stretch in the Q/N-repeats destabilized their assembly as amyloid. Interestingly, in the absence of any OPRs all the constructs including RepA-WH1 repeats (R0 + WH1-R1-5) gave a nearly WT phenotype, indicating that, in these chimeras, amyloids were successfully built with little interference between the RepA-WH1 and the Sup35p moieties (**Figure 2A**). In adenine-deficient media, all chimeras supported yeast growth, i.e., they read-through *ade1-14* thus restoring a functional pathway for adenine synthesis, albeit R5 + WH1-R1 led again to a poor phenotype (**Figure 2B**).

When, after several passages through liquid rich medium, yeast carrying the chimeras were plated on ¼YPD agar (**Figure 2C**) red sectored colonies appeared if the number of WH1 peptide repeats was ≤2, indicating that these were unstable chimeric prions. The chimeric prions including ≥3 WH1 repeats, which we termed [*REP-PSI*^+^], exhibited two clearly different phenotypes, namely white and pink colonies (**Figure 2D**, top). To explore the mitotic stability of both phenotypes, single colonies of each kind were subcultured and plated again on ¼YPD. White colonies showed a remarkable stability (frequency of conversion to red ≈10^-6^, matching that described for [*PSI*^+^]; as reviewed in Liebman and Chernoff, 2012; **Figure 2D**, left), whereas light pink colonies were very unstable, giving rise with high frequencies to dark pink (≈2 × 10^-1^) and red (≈10^-2^), or reverting to white (≈3 × 10^-3^), colonies (**Figure 2D**, right). Tentatively, the white phenotype was associated to a strong prion variant, *s*[*REP-PSI*^+^], the two pink tones to weak prion variants, *w1* and *w2*[*REP-PSI*^+^] (light or dark pink, respectively), and the red colonies to cured (i.e., having lost the prion phenotype) [*rep-psi*^-^].

### [*REP-PSI*^+^] Prion is Infectious and Templates on Sup35p a New Weak, Unstable Variant, [*PSI*^+^]*^WH*1*^*

**FIGURE 3:**
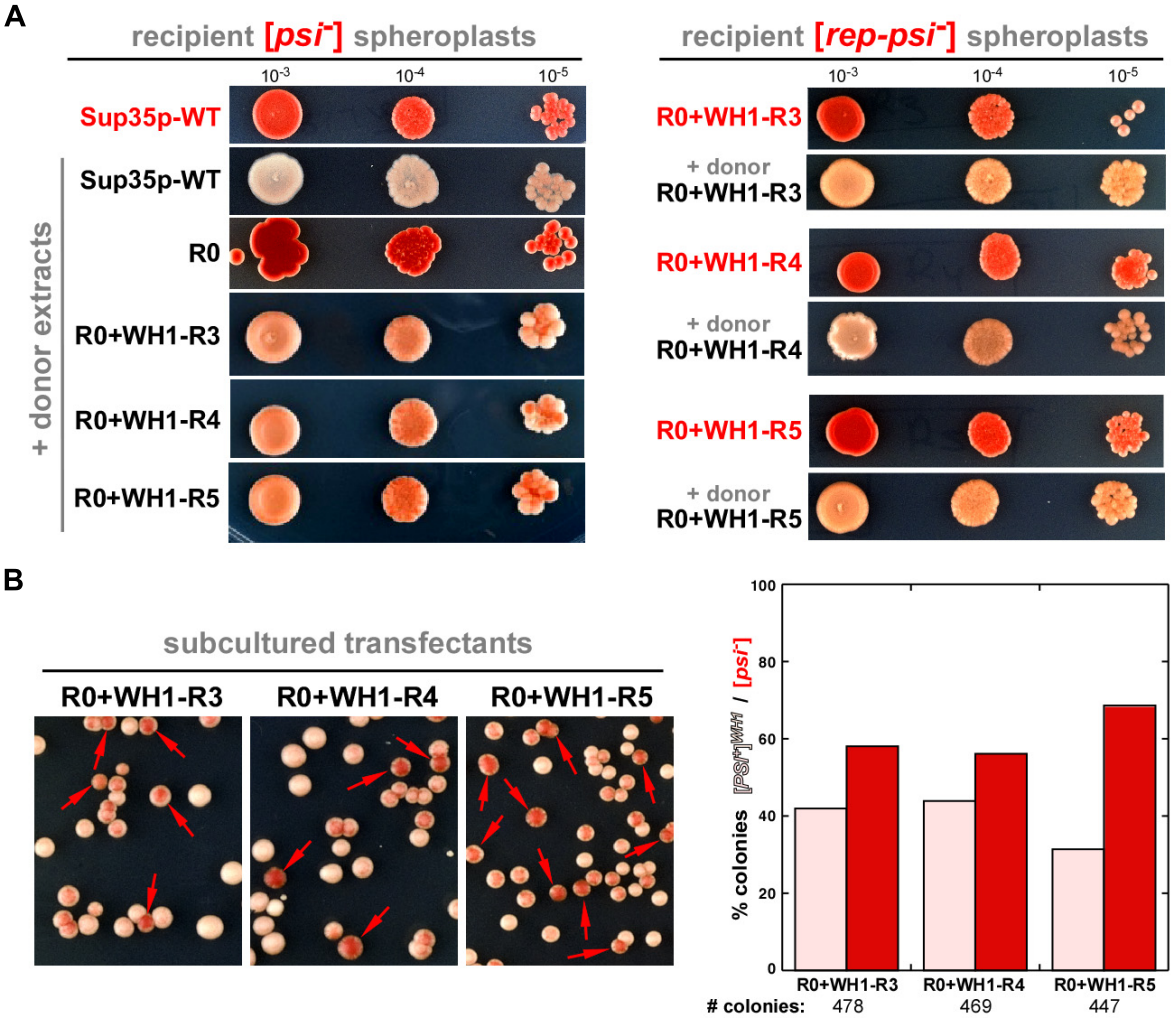
**[*REP-PSI*^+^] aggregates template a prion phenotype on recipient [*psi^-^*] and [*rep-psi^-^*] cells. (A)** Yeast spheroplasts prepared from red [*psi^-^*] (left) or [*rep-psi^-^*] (right) colonies that had spontaneously lost the prion phenotype (red labels) were transformed with extracts from either [*PSI*^+^] or [*REP-PSI*^+^] cells (black labels). Ten-fold serial dilutions of cultures from the transformed colonies, starting with OD_600_ = 0.001, were plated on ¼YPD agar. [*REP-PSI*^+^] extracts were able to confer a light pink, weak [*PSI*^+^]-like phenotype to the recipient cells expressing Sup35p-WT (left), or a [*REP-PSI*^+^] phenotype to those expressing the indicated R0 + WH1-R3-5 chimeras (right). **(B)** Cells expressing Sup35p-WT and transformed with R0 + WH1-R3-5 [*REP-PSI*^+^] aggregates (**A**, left) were diluted 10^-4^ in non-selective YPD medium and then plated on ¼YPD agar. The high frequency of appearance of red colonies (arrows) besides the pink clones revealed the instability of a new prion variant, [*PSI*^+^]*^WH*1*^*, templated by the chimeras on Sup35p-WT. Right: a quantitative estimation of stability of the new prion variant, in the form of a histogram display of the mean values for the ratio between the number of pink [*PSI*^+^]*^WH*1*^* and red [*psi^-^*] colonies from two independent transformation rounds of the R0 + WH1-R3-5 aggregates with subsequent growth in YPD.

Yeast spheroplasts can be transformed with prion particles, either assembled *in vitro* or extracted from cultured cells (Tanaka, 2010). Spheroplasts prepared from red colonies, thus expressing either Sup35p-WT or the WH1-R3-5 chimeras in their non-prion form ([*psi*^-^] or [*rep-psi*^-^], respectively), were transformed with protein extracts from cells also carrying the chimeras but grown from white colonies, thus with a [*REP-PSI*^+^] phenotype. When the epigenetic phenotype of the transformants was assayed (**Figure 3A**), all colonies showed the white or the light pink stop codon read-through phenotype characteristic of the incoming prion aggregates (Sup35p-WT or the chimeras, respectively). The demonstration of the ‘infectivity’ of the R0 + WH1-R3-5 chimeras through the transformation of their aggregated forms qualifies [*REP-PSI*^+^] as a new synthetic yeast prion. However, after serial sub-culturing without selective pressure, in those clones expressing Sup35p-WT and cross-transformed with the WH1-R3-5 [*REP-PSI*^+^] chimeras, besides the pink phenotype of the incoming aggregates, red [*psi*^-^] colonies appeared frequently (**Figure 3B**). These results indicated that the WH1-R3-5 chimeras can template their conformation on Sup35p-WT to generate a new very weak prion strain (or ensemble of weak strains) that we have named [*PSI*^+^]*^WH*1*^*.

### [*REP-PSI*^+^] Prion Assembles Amyloids Larger than [*PSI*^+^]

**FIGURE 4:**
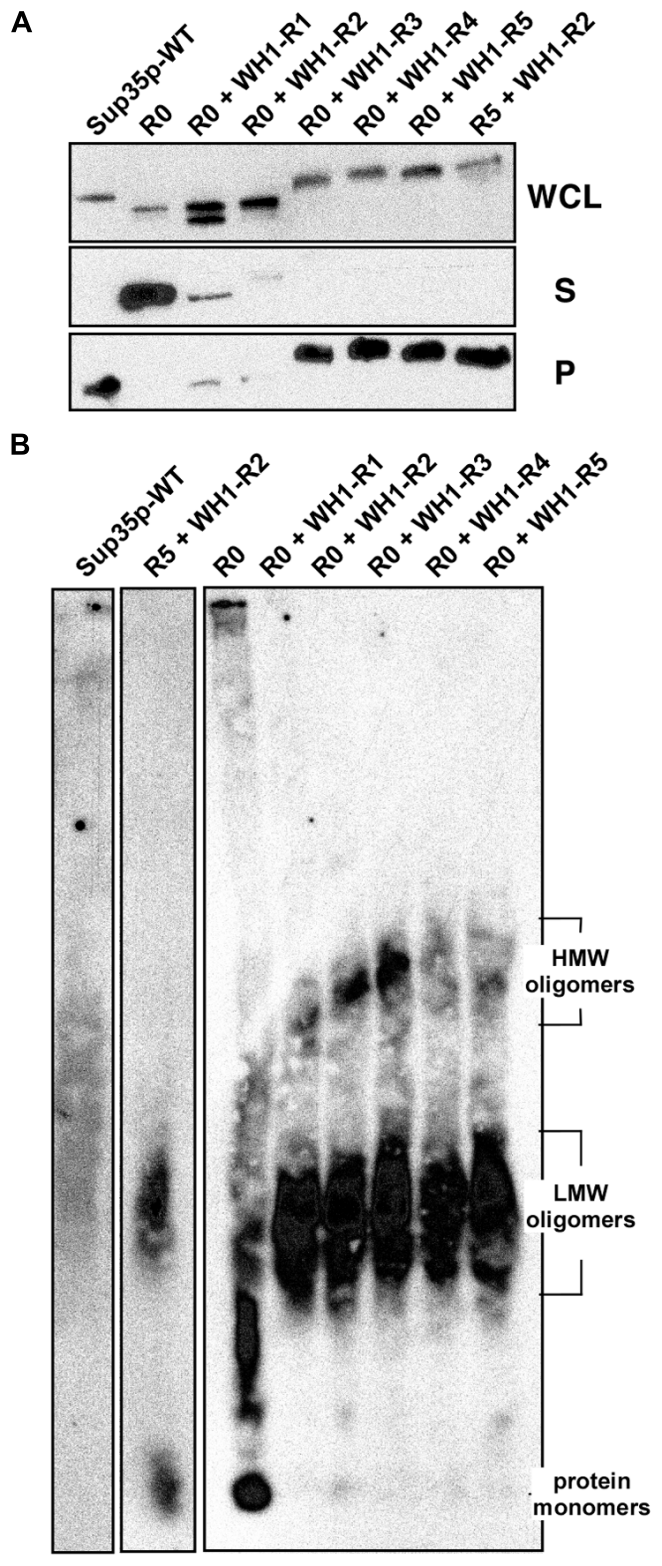
**[*REP-PSI*^+^] prions assemble amyloid oligomers larger than [*PSI*^+^]. (A)** The soluble (S) and the aggregated (P) protein fractions in whole cellular lysates (WCL) from yeast overproducing the indicated constructs were separated by ultracentrifugation. Sup35p and the R5 + WH1-R2 and R0 + WH1-R3-5 chimeras preferentially aggregate. **(B)** In extracts from cells expressing the chimeras, SDD-AGE resolves two populations of detergent-resistant amyloid aggregates, with distinct average molecular weights (Low-MW and High-MW).

Biochemical analysis of the solubility of the engineered chimeric proteins, performed upon their overexpression from the *GAL1* promoter, showed that constructs R0 + WH1-R3-5 aggregated massively, whereas R0 + WH1-R1-2 were proteolytically more unstable (**Figure 4A**). Regarding the extra, higher mobility band observed in the SDS-PAGE of the whole cellular lysates for R0 + WH1-R1, including a single WH1 hydrophobic repeat in the context of the Sup35p Q/N-rich sequences might generate instability in the natural beta-arcades built by the prion, which will thus become proteolysis-prone. Following this speculation, that higher mobility band would correspond to molecules of the chimera that have lost the bit N-terminal to the single engineered WH1 repeat. The R0 + WH1-R1-2 chimeras were further degraded during the manipulation of the cell lysates for ultracentrifugation analysis, in spite of performing the experiment at low temperature and supplying the samples with protease inhibitors. Semi-denaturing detergent agarose gel electrophoresis (SDD-AGE), a technique for the detection of amyloid aggregates in a broad range of sizes (Bagriantsev et al., 2006), was carried out by immediately running in the gel the WCL fractions under conditions that should denature proteases, thus reducing degradation of the R0 + WH1-R1-2 chimeras to a minimum. SDD-AGE revealed (**Figure 4B**) that the chimeras form two populations of aggregates according to their electrophoretic mobilities. The species with higher molecular weights were evident in R0 + WH1-R1-5, showing sizes in direct correlation with the increasing number of RepA-WH1 repeats, but were barely detectable for Sup35p-WT, R0 or the construct carrying R5 + WH1-R2. This result points to the construction of a distinct type of assembly by the chimeras in which the hydrophobic repeats have completely replaced the Q/N-OPRs. The presence of very high molecular weight species in SDD-AGE has been described in [*PSI*^+^] variants resistant to Hsp104 chaperone-promoted shearing, thus resulting in weak prion phenotypes with low mitotic stabilities (Derdowski et al., 2010; Alexandrov et al., 2012).

### RepA-WH1 Repeats in [*REP-PSI*^+^] Alter the Structure of the Amyloids Assembled by the NM-Domains

**FIGURE 5:**
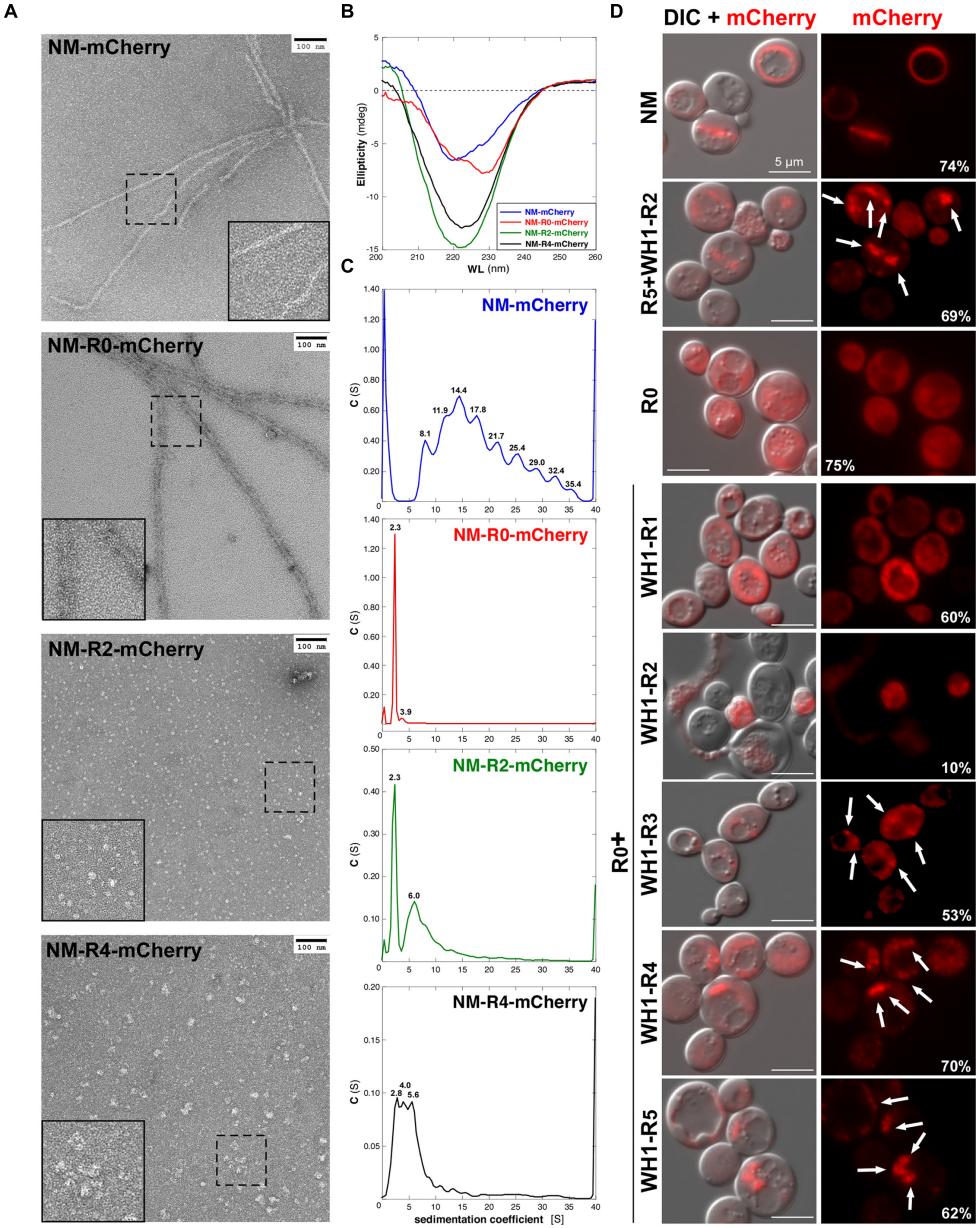
**Chimeric NM domains from [*REP-PSI*^+^] fused to the mCherry reporter form oligomeric assemblies. (A)** Purified His_6_-tagged NM-mCherry fusions (15 μM) were assembled *in vitro* and visualized by transmission electron microscopy. Insets are twofold magnifications of areas within dashed boxes. RepA-WH1 repeats drive the assembly of the NM-mCherry proteins into particles whose sizes increase with the number or repeats. **(B)** Circular dicroism (CD) spectra of the purified, preassembled NM-mCherry proteins (15 μM) showed an increase in β-sheet structure (broad minima at ≈220 nm) for the R0 + WH1-R2/4 chimeras.**(C)** Sedimentation velocity experiments were performed in an analytical ultracentrifuge with the same purified NM-mCherry proteins (10 μM) studied in **(A,B)**. The analyses indicate poly-dispersed aggregation for the chimeras (i.e., multi-peaked profiles), with a direct correlation between the number of RepA-WH1 repeats and the sedimentation coefficient values/number of peaks. However, the profiles for the R0 + WH1-R2/4 chimeras were simpler than that for the NM-Sup35 fusion, reflecting the ability of the latter to assemble fibers **(A)**. **(D)** Epifluorescence microscopy imaging of yeast cells expressing the chimeric NM-mCherry fusions. Exposure times: 200 ms (Sup35-WT, R0, R0 + WH1-R1), 600 ms (R5 + WH1-R2, R0 + WH1-R3-5), and 2 s (R0 + WH1-R2). ND filter: ¼ Left: superposition of the DIC and fluorescence images. Chimeras carrying R0 + WH1-R3-5 form foci (arrows) whose sizes increase with the number of WH1 repeats, whereas WH1-R1 is dispersed through the cytoplasm and R0 + WH1-R2 is cytotoxic (cell lysis). The fraction of cells expressing the chimeras (i.e., those fluorescence-labeled) is indicated (%); 200–400 cells of each type were counted.

The results above pointed to a singular structural arrangement in [*REP-PSI*^+^] for the hydrophobic RepA-WH1 OPRs within the flanking polar Q/N-rich sequences, which remained unaltered from the Sup35p N-domain. The N and M domains (**Figure 1A**) in the [*REP-PSI*^+^] chimeras were then fused to the monomeric red fluorescent protein mCherry and expressed in *E. coli*. Those expressed at sufficiently high levels were purified and their assembly morphologies (**Figure 5A**), average secondary structure compositions (**Figure 5B**), and association states (**Figure 5C**) were physically analyzed. NM-mCherry and NM-R0-mCherry controls were able to assemble into fibers under standard conditions (Giraldo, 2007; Fernández-Tresguerres et al., 2010; **Figure 5A**). However, NM-mCherry fusions carrying WH1-R2 or WH1-R4 did not form fibers, but irregular oligomers whose average sizes (≤10 and ≈25 nm, respectively), directly correlated with the number of hydrophobic repeats. Such oligomers were compatible with the smaller aggregates detected by means of SDD-AGE for the [*REP-PSI*^+^] chimeras (**Figure 4B**).

Circular dichroism (CD) analysis of the purified proteins (**Figure 5B**) indicated a net increase in β-sheet structure (broad band at ≈220 nm) for NM-R2/4-mCherry compared with NM-mCherry, whereas NM-R0-mCherry spectrum resembled the spectra of Q/N-rich peptides when forming coiled-coils (red-shifted band at >225 nm; Fiumara et al., 2010). Since sedimentation velocity experiments (**Figure 5C**) showed that purified NM-R0-mCherry was a monomer (*s* = 2.3 S), such coiled-coil should be intramolecular. On the contrary, poly-dispersed aggregation was evident as multiple peaks with increasing sedimentation coefficients for NM-R2/4-mCherry and the WT control NM-mCherry. These *in vitro* experiments indicated that the chimeric [*REP-PSI*^+^] prions, carrying hydrophobic OPRs of bacterial origin, assemble as amyloid oligomers rather than fibers, as Sup35p/[*PSI*^+^] does.

### [*REP-PSI*^+^] Prion Aggregates as Perivacuolar Foci *In Vivo*

The NM-mCherry fusion proteins were then expressed in yeast from a Gal-inducible plasmid (**Figure 5D**). When the N domain carried the Sup35p wild-type sequence, the characteristic ring-like aggregates appeared in the cytoplasm (Tyedmers et al., 2010), whereas if it lacked all the OPRs (NM-R0-mCherry) fluorescence labeling was found diffused. If the constructs included the N domain of the distinct [*REP-PSI*^+^] chimeras, aggregation appeared as multiple dots/foci whose sizes increased with the number of RepA-WH1 repeats. They were disposed around the vacuole (IPOD compartment), as previously described for mature NM-GFP amyloids (Tyedmers et al., 2010). Interestingly, the fraction of yeast cells expressing the NM-R2-mCherry chimera was significantly reduced, and the lysis of many cells became evident. It has been proposed that the cellular toxicity of hydrophobic amyloidogenic peptides is linked to their ability to assemble as oligomeric pores upon insertion into lipid bilayers (reviewed in Butterfield and Lashuel, 2010). It might be the case that NM-R2-mCherry, lacking the ability to further assemble into a stable parallel superpleated β-structure (**Figure 1B**), would have a preference for membrane targeting.

## Discussion

In this work, we have generated chimeras (**Figure 1**) by replacing the polar OPRs in Sup35p with tandem repeats of the hydrophobic amyloidogenic stretch found in the bacterial prionoid RepA-WH1 (Giraldo, 2007; Gasset-Rosa et al., 2008a, 2014). The resulting synthetic [*REP-PSI*^+^] prions are functional in yeast, generating both a strong [*PSI*^+^]–like phenotype and various weak phenotypes (**Figures 2 and 3**), which are compatible with a cloud of prions strains (Bateman and Wickner, 2013). These chimeric prions assemble themselves *in vitro* as discrete size particles (**Figure 5**), which probably would suffice to act as competent propagons *in vivo*.

Our results suggest that cross-seeding of [*REP-PSI*^+^] through conformational templating, since the wild-type OPRs were absent from the chimeras, must be exerted by the flanking Q/N-rich sequences in the N-terminal domain that come from Sup35p-WT. This is compatible with findings showing that mutants including hydrophobic and/or aromatic residues at the N-terminus (residues 1–40) of Sup35p enhance [*PSI*^+^] nucleation, whereas aromatic side-chains at the OPRs promote chaperone-mediated propagation (Toombs et al., 2010, 2011; Alexandrov et al., 2012; Gonzalez-Nelson et al., 2014; MacLea et al., 2015). It is noteworthy that such studies were performed by mutating a single OPR out of five in Sup35p, yielding constructs somehow analogous to our unstable R5 + WH1-R1-2 chimeras, whereas in this work we have built a complete assembly made of up to five hydrophobic repeats (R0 + WH1-R1-5). According to the results discussed here, there seems to be in [*REP-PSI*^+^] a minimum threshold of three hydrophobic WH1 repeats in order to build a hydrophobic spine in the β-arcades sufficiently stable to surpass the control exerted by the surrounding natural polar sequences on prion nucleation and propagation. Thus, the engineered superpleated β-structure in [*REP-PSI*^+^] seems to behave as an orthogonal synthetic module functional in prion propagation, in the sense proposed by Toombs et al. (2012). It has also been described that point mutations replacing polar Q/Y residues by charged Lys in OPRs positions confronted in the β-arcades, thus leading to electrostatic repulsion, result in new prion variants, [*PSI*^+^]*^M1-M5^*, some of which are unstable, and template on Sup35p-WT a non-epigenetically heritable conformation (Bondarev et al., 2013, 2014). The ‘prion no more’ mutant G58D, which affects the second OPR (R2) in Sup35p, can be incorporated in WT aggregates, leading to increased frequency of fragmentation, thus compensating for deficiencies in propagation of weak [*PSI*^+^] variants but curing, as dominant-negative, strong prion variants (DiSalvo et al., 2011; Verges et al., 2011). We have shown here that even more extensive engineering of the OPRs can give way to a new prion, [*REP-PSI*^+^], that templates on Sup35p a new weak variant, [*PSI*^+^]*^WH*1*^*. Conversely, during the initial expression of the WH1-R1-5 chimeras from plasmids, the [*PSI*^+^] prion resident in the yeast cells, before the displacement of its encoding (*SUP35*) vector, could influence through templating the compatibility and selection of the distinct [*REP-PSI*^+^] variants described in this work. Cross-seeded aggregation between distinct RepA-WH1 variants has also been reported in bacteria (Molina-García and Giraldo, 2014). The possible contribution of the [*PSI*^+^]-ancillary prion Rnq1p/[*PIN*^+^] (Sharma and Liebman, 2013) to [*REP-PSI*^+^] nucleation will require further studies.

Interestingly, the R0 + WH1-R3-5 chimeras build oligomers rather than fibers, as Sup35p does: most probably, having a large hydrophobic beta-arcade inserted in the polar Q/N-rich prion domain will impose a hindrance to the assembly of long, structurally regular fibers. Similarly, the natural, non-Q/N-rich prion [*GAR*^+^] (Brown and Lindquist, 2009), which has recently been described to overcome glucose catabolite repression in *Saccharomyces cerevisiae* and other yeast (Jarosz et al., 2014), does not assemble as fibers but as oligomeric aggregates, as described here for the [*REP-PSI*^+^] chimeras. In yeast, under normal conditions, binding of Ssa1p (an Hsp70 chaperone) to Sup35p targets Hsp104p to [*PSI*^+^] aggregates for the generation of prion seeds (Winkler et al., 2012). Sup35p alleles defective in OPRs generate prions, such as [*PSI*^+^]^Δ*22/69*^ (Borchsenius et al., 2001), that also build large aggregates behaving as unstable, weak prion variants (Tanaka et al., 2006; Derdowski et al., 2010). When the Q/N-rich OPRs in *S. cerevisiae* Sup35p were replaced by heterologous non-Q/N sequences from other yeast species, the propagation of the resulting chimeric prion became independent on Hsp104p (Crist et al., 2003). The region between the OPRs and the initial residues in the medium (M) domain is the target recognized by Hsp104p in the Sup35p amyloids (Frederick et al., 2014). Besides this, the intermolecular contacts characteristic of weak [*PSI*^+^] variants exhibit an increased dependence on Hsp70s for Hsp104p-driven shearing, compared with those found in strong variants (DeSantis and Shorter, 2012). The propagation of [*GAR*^+^] is independent of the activity of the Hsp104 disaggregase, probably because the oligomeric nature of this prion allows for diffusion-driven propagation, but this becomes strictly dependent on Hsp70 (Brown and Lindquist, 2009; Jarosz et al., 2014). Interestingly, the bacterial Hsp104p orthologue, ClpB, does not contribute either to the propagation of the RepA-WH1 prionoid in *E. coli*, a function relying mainly on the Hsp70 chaperone DnaK (Gasset-Rosa et al., 2014). DnaK conformationally selects for an amyloid variant of RepA-WH1 with reduced toxicity and generates relatively small oligomeric particles, readily diffusible to the progeny (Gasset-Rosa et al., 2014). In the case of [*REP-PSI*^+^], the contribution of chaperones to its propagation in yeast remains to be explored.

Heterologous model systems have made fundamental contributions to the understanding of prion propagation. The expression of the yeast prion [*PSI*^+^] in bacteria has revealed that Sup35p aggregates as inclusion bodies which retain the ability to nucleate distinct strains (Garrity et al., 2010; Espargaró et al., 2012), and that, as in its original host, it still needs nucleation by [*PIN*^+^] (Garrity et al., 2010) and depends on Hsp104 for propagation (Yuan et al., 2014). In addition, [*PSI*^+^] has also been propagated in mammalian cells in culture, exhibiting the hallmarks of cytoplasmic inheritance (Krammer et al., 2009; Hofmann et al., 2013). The synthetic [*REP-PSI*^+^] prion, by expanding the repertoire of [*PSI*^+^] variants and rewiring amyloidogenic parts of Sup35p with alien, non-Q/N-rich sequences of bacterial origin, is a proof of concept for the feasibility of generating new phenotypic traits in prions. Engineering the consortium between [*REP-PSI*^+^] and its possible chaperone modulators will surely enable new trends in yeast epigenetics.

## Conflict of Interest Statement

The authors declare that the research was conducted in the absence of any commercial or financial relationships that could be construed as a potential conflict of interest.
